# The first complete genome sequence of iris yellow spot virus from South America and comparative analysis with isolates of different geographic origin

**DOI:** 10.1007/s00705-026-06718-x

**Published:** 2026-08-11

**Authors:** Rosana Blawid, Edivânio Rodrigues de Araújo, Leandro Delalibera Geremias, Dennis Knierim, Priscila Duarte Ramirez, Paolo Margaria

**Affiliations:** 1 Department of Agronomy, Laboratory of Plant Virology, Federal Rural University of Pernambuco, Recife, Brazil; 2 Epagri- Experimental Station of Ituporanga, Santa Catarina, Brazil; 3 Plant Virus Department, Leibniz Institute DSMZ-German Collection of Microorganisms and Cell Cultures GmbH, Inhoffenstraße 7B, Braunschweig, Germany

## Abstract

We report the first complete nucleotide sequence of an isolate of iris yellow spot virus (IYSV; *Orthotospovirus iridimaculaflavi*) from Brazil. Isolate Br-25 was collected in 2025 in Imbuia, Santa Catarina, from symptomatic onion scapes. The genome of IYSV-Br-25 consisted of L, M and S genomic RNAs of 8879, 4866 and 3223 nt, respectively. Comparison of the L segment nucleotide (nt) sequence showed identities ~96% to most of the available L segment sequences from IYSV isolates of different geographic origin, with the exception of isolate DSMZ PV-0528, which showed 89.0% nt identity. The M and S segment nt sequences showed highest identity to an isolate recently reported from sugarbeet in Idaho, USA. *In silico*-generated *Hinf*1 digestion profile assigned the isolate to the previously defined IYSV_BR_ genotype. This work provides valuable novel information on the genetic diversity of IYSV.

**Supplementary Information:**

The online version contains supplementary material available at https://doi.org/10.1007/s00705-026-06718-x.

Thirty-six species are currently recognized within the genus *Orthotospovirus*, the sole genus in the family *Tospoviridae* (https://ictv.global/msl, MSL41.v1). Iris yellow spot virus (IYSV; species *Orthotospovirus iridimaculaflavi*) is an economically important virus on onion and bulb crops in many regions of the world [1–6]. In addition to cultivated alliaceous crops, IYSV has been reported to infect wild *Allium* species, weeds and ornamental plants [2, 7]. Early reports of this virus date back to the 1990s [8–11], and the first molecular and serological characterization studies were conducted on an isolate from *Iris hollandica,* from which the virus takes the name [10]. The virus genome is segmented and composed of three RNA molecules, referred to as large (L), medium (M) and small (S). The L RNA encodes the RNA-dependent RNA polymerase (RdRp) in viral complementary (vc) sense [12]. The M RNA has an ambisense coding strategy and codes for the G_N_/G_C_ glycoproteins precursor in the vc sense and a non-structural protein (NSm) in the viral (v) sense [13]. The S RNA also exhibits an ambisense coding strategy, with the coat protein (N) encoded in the vc sense and a non-structural protein (NSs) encoded in the v sense [10, 11].

A recent sequence mining in GenBank has revealed availability of only a few IYSV genome sequences with complete or near-complete information. The coding-complete sequence is available only for an Italian isolate (GenBank accession no. OR526469, OR526470, OR526471) [14]; https://archiplavit.to.cnr.it/] and for an isolate from the United States of America (USA) (PQ246270, PQ246271, PQ246272) [15]. The GenBank nuclear core (NC) reference sequences are assigned to an isolate from USA (L segment sequence, NC_029799) [12] and The Netherlands (M and S segment sequences: NC_038232, NC_029800, respectively) [10, 13]. The sequences of the three genomic segments for an isolate from Zimbabwe are available too (OQ985364, OQ985365, OQ985366); however, the S segment sequence contains undetermined residues in the intergenic region and terminal ends are not determined for any of the segments [16]. Genome sequences of other IYSV isolates of worldwide origin (India, Iran, USA) are available, however none of them covering the whole genome information. Most represented are partial sequences covering the N gene, of which over 170 sequences are currently available in the GenBank public database.

In this study, we provide the first complete genome sequence of an IYSV isolate from South America. In the state of Santa Catarina (Brazil), IYSV was first detected in 2017, and exhibited an occurrence frequency of 31.4% in samples showing suspected symptoms during the 2017–2019 crop seasons [17]. Plant material for this study was collected in 2025 in Imbuia, Santa Catarina, from volunteer onion plants nearby a commercial field, showing light green, diamond-shaped spots on the scapes (Fig. 1).

**Fig. 1:**
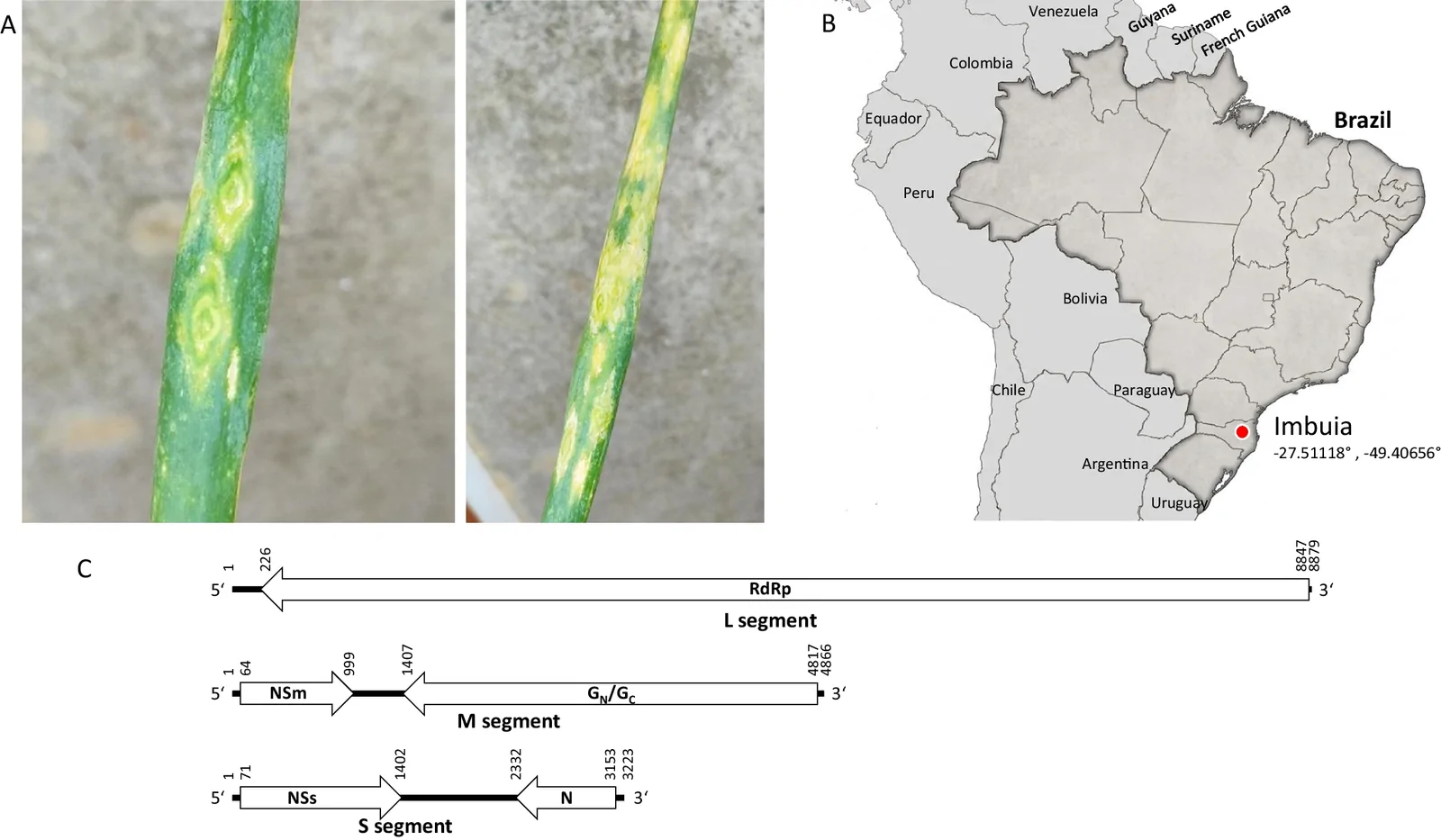
**A** Symptoms observed on two independent onion scapes at the time of the survey in 2025. **B** Geographic map showing the location of samples collection. **C** Schematic depiction of the genome organization of IYSV isolate Br-25 characterized in this study

The presence of IYSV was initially confirmed using IYSV ImmunoStrips (Agdia Inc., Elkhart, IN, USA). To reconstruct the genome sequence of IYSV isolate Br-25, a library was constructed from total RNA extracted from symptomatic leaves of an individual onion plant, using a NEBNext UltraExpress® RNA Library Prep Kit (New England Biolabs, USA), including a ribosomal RNA-depletion treatment according to the QIAseq FastSelect –rRNA Plant Kit (Qiagen, Germany). Following quantification and quality control, the library was subjected to high-throughput sequencing (HTS) on an Illumina NextSeq2000 platform at Leibniz Institute DSMZ (Braunschweig, Germany), resulting in 41,442,060 raw reads (Suppl. Table 1), which were processed in Geneious Prime v. 2026.0.2 according to the pipeline developed in house for virus discovery. In brief, the reads were paired, trimmed using BBDuk v. 38.84, normalized, merged, and aligned to plant-related sequences (Suppl. Table 2) using the Geneious mapper, allowing 20% maximum mismatches per read. The unmapped reads were *de novo* assembled using the Geneious assembler implemented in Geneious Prime, allowing 10% maximum mismatches per read. The resulting contigs were screened by BLAST [18] against a custom virus/viroid reference database. BLASTn alignment of the generated contigs identified sequences assigned to Allium delpartitivirus, Allium deltapartitivirus 2, Allium cepa amalgavirus 1, and IYSV (Table S1). IYSV was further characterized in this work to obtain the complete genome information (Fig. 1).

Mean coverage of L, M and S genome segments was 1382.4x, 1473.0x and 2433.0x, respectively. The L segment consisted of 8879 nt, and contains an ORF of 8622 nt in the vc sense (position 8847-226), coding for the putative RdRp protein of 2873 aa, with a predicted molecular mass of 331.3 kDa. The 3´ and 5´ UTRs consisted of 32 and 225 nt, respectively. The M segment consisted of 4866 nt with short 5´ and 3´ UTR ends of 63 and 49 nt, respectively; it encodes the putative NSm protein in the v sense (nt position 64–999) and the G_N_/G_C_ glycoprotein precursor in the vc sense (position 4817-1407). The two ORFs are separated by an intergenic region of 407 nt. The bicistronic S segment consisted of 3223 nt and encodes the NSs protein in position 71–1402 nt, and the N protein in position 3153-2332, with an intergenic region of 929 nt. The S segment 3´ and 5´ UTRs are both of 70 nt in length.

Pairwise alignment comparisons of the L gene sequence with other isolates of worldwide origin using Sequence Demarcation Tool (v. 1.3) software [19] showed nt identities of about 96% to most (four out of five) of the available sequences from different continents, with the exception of isolate DSMZ PV-0528, which shared 89.0% common nucleotides (Table 1).

**Table 1:** Sequence identity at nucleotide (nt) and amino acid (aa) level of iris yellow spot virus (IYSV) isolate Br-25 versus other IYSV sequences available in the NCBI GenBank public database, as determined using Sequence Demarcation Tool (v. 1.3) software [19]

Isolate	Origin	GenBank accession^			Collection date	% identity (nt)			% identity (nt/aa)				
		L segment	M segment	S segment		L segment	M segment	S segment	RdRp	G_N_/G_C_	NSm	N	NSs
Bv_Parma	USA	PQ246270	PQ246271	PQ246272	2022	96.4	98.1	97.4	96.4/98.7	98.7/99.4	97.3/99.7	97.8/97.1	97.9/98.4
IYSV	USA	**NC_029799**	n.d.	n.d.	2008	96.1	-	-	96.1/98.3	-	-	-	-
DSMZ PV-0528	Unknown	**OP357937**	OP357938*	**OP357939**	before 26.11.1998	89.2	-*	88.8	89.0/95.6	-	89.9/94.9	88.3/90.5	90.8/96.4
IYSV_ZIM_L	Zimbabwe	OQ985364°	OQ985365°	OQ985366°**	2020	95.9	95.8	90.2	95.6/98.3	96.0/97.7	95.7/99.0	93.3/92.6	96.1/97.5
PLAVIT-Cip6	Italy	OR526469	OR526470	**OR526471**	2009	96.3	98.1	96.4	96.3/98.9	98.4/98.9	97.2/97.7	96.8/96.0	97.7/98.0
DOGR	India	n.d.	KM035409	KJ868797	2014	-	87.6	88.0	-	88.3/90.9	86.9/89.1	87.1/89.0	91.1/95.3
IYSV	The Netherlands	n.d.	**NC_038232**	**NC_029800**	1992	-	88.8	89.0	-	88.8/92.3	89.9/94.2	88.6/90.1	91.0/96.6
Le1M	Iran	n.d.	n.d.	**MG065699**	2015	-	-	88.0	-	-	-	88.3/91.6	90.3/95.3
IYSV	USA	n.d.	FJ361359	n.d.	Unknown	-	98.0		-	98.2/99.0	97.9/99.7	-	-

^*^M segment sequence is available in GenBank, however described as defective-interfering form

^**^Undetermined nt residues are present in the sequence of the intergenic region

°sequence contains wobbles

^GenBank accessions in bold refer to full-length segment sequences

n.d.: not determined

At aa level, identities ranged from 95.6% to 98.9% (Table 1). Analysis of the full-length sequence of the M segment showed identities values in the range 95.8% to 98.1% with isolates from the USA, Italy and Zimbabwe (Table 1). Of interest the percentages dropped to 87.6% and 88.8% against the sequences of an isolate from India and the GenBank NC reference isolate from The Netherlands, respectively. This scenario was reflected as well when investigating the G_N_/G_C_ sequences at nt and aa level, with ranges 88.3%/98.7% and 90.9%/99.4%, respectively. Regarding the NSm gene, identity values above 95.7% were observed for the isolates from the USA, Italy and Zimbabwe, while against the NC reference sequence from The Netherlands, an Indian isolate and the DSMZ isolate, the values ranged from 86.9% to 89.9%. At aa level, the isolate from India was the only one with NSm aa identity below 90% to Br-25. Analysis of the S segment revealed pairwise nt identities in the range 88.0%−97.4%, with the isolates from India and Iran showing the lowest identity, and the isolate Bv_Parma from the USA showing the highest identity. At the N gene level, isolate Br-25 showed identities in the range 93.3%/97.8% with isolates from Italy, USA and Zimbabwe, and 87.1%/88.6% with isolates from Iran, The Netherlands, and India. The same profile was observed for the NSs at both nt and aa level.

Based on previous studies on IYSV sequence diversity using the N gene [20, 21], we investigated *in silico* the predicted profile generated by *Hinf*1 restriction enzyme digestion. Eight digestion sites were predicted (at nt 27, 44, 223, 259, 567, 727, 746, 797), with a largest fragment of 308 nt in size (not shown), assigning Br-25 to the IYSV_BR_ genotype [20]. Alignment against the whole dataset of N gene sequences from GenBank (*n=*173; accessed on 09.03.2026) showed that the N gene of isolate Br-25 had lowest sequence identity (84.1%) to an isolate from Slovenia (GenBank accession AY377428) and Australia (acc. AY345226), and highest identity (98.3%) to an isolate from Bosnia and Herzegovina (acc. KF733020) and from Indonesia (acc. PV543594). Phylogenetic analysis using the whole collection of N gene sequences showed that isolate Br-25 grouped within the previously defined IYSV_NL_ cluster (Suppl. Fig. 1). Notably, the type isolate of IYSV_BR_ (AF067070) grouped in the IYSV_NL_ clade [20]. Recombination analysis on the N gene sequences using RDP software (v. 4.101) [22] did not predict the occurrence of putative recombination events according to criteria defined previously [23].

Given the limited number of IYSV sequences available in GenBank and the absence of a sequence from the South-American continent, our study significantly contributes novel information on IYSV genetic diversity at worldwide scale.

## Supplementary Information

Below is the link to the electronic supplementary material.

Supplementary file1 (PPTX 69.3 KB)

Supplementary file2 (XLSX 14.0 KB)

Supplementary file3 (XLSX 15 KB)

## Data Availability

The high-throughput sequencing dataset generated during the current study is available from the Plant Virus Department - Leibniz Institute DSMZ on reasonable request. The complete sequences of the L, M and S segment of IYSV Br-25 are available in GenBank under accession numbers PZ293830, PZ293831, PZ293832.
